# Machining of Titanium Metal Matrix Composites: Progress Overview

**DOI:** 10.3390/ma13215011

**Published:** 2020-11-06

**Authors:** Cécile Escaich, Zhongde Shi, Luc Baron, Marek Balazinski

**Affiliations:** 1 Department of Mechanical Engineering, Polytechnique Montréal, 2500 Chemin de Polytechnique, Montréal, QC H3T 1J4, Canada; luc.baron@polymtl.ca (L.B.); Marek.Balazinski@polymtl.ca (M.B.); 2 Aerospace Manufacturing Technology Center, National Research Council Canada, 5145 Ave. Decelles, Montréal, QC H3T 2B2, Canada; Zhongde.Shi@cnrc-nrc.gc.ca

**Keywords:** titanium metal matrix composites, material removal mechanisms, tool wear, surface quality

## Abstract

The TiC particles in titanium metal matrix composites (TiMMCs) make them difficult to machine. As a specific MMC, it is legitimate to wonder if the cutting mechanisms of TiMMCs are the same as or similar to those of MMCs. For this purpose, the tool wear mechanisms for turning, milling, and grinding are reviewed in this paper and compared with those for other MMCs. In addition, the chip formation and morphology, the material removal mechanism and surface quality are discussed for the different machining processes and examined thoroughly. Comparisons of the machining mechanisms between the TiMMCs and MMCs indicate that the findings for other MMCs should not be taken for granted for TiMMCs for the machining processes reviewed. The increase in cutting speed leads to a decrease in roughness value during grinding and an increase of the tool life during turning. Unconventional machining such as laser-assisted turning is effective to increase tool life. Under certain conditions, a “wear shield” was observed during the early stages of tool wear during turning, thereby increasing tool life considerably. The studies carried out on milling showed that the cutting parameters affecting surface roughness and tool wear are dependent on the tool material. The high temperatures and high shears that occur during machining lead to microstructural changes in the workpiece during grinding, and in the chips during turning. The adiabatic shear band (ASB) of the chips is the seat of the sub-grains’ formation. Finally, the cutting speed and lubrication influenced dust emission during turning but more studies are needed to validate this finding. For the milling or grinding, there are major areas to be considered for thoroughly understanding the machining behavior of TiMMCs (tool wear mechanisms, chip formation, dust emission, etc.).

## 1. Introduction

Titanium and its alloys have been the subject of significant research interest in different fields. The unique properties of these alloys such as high specific strength, lightweight, chemical resistance, and biocompatibility make them ideal candidates for biomedical, automotive, petrochemical, and aerospace industries [1,2,3]. Titanium metal matrix composites (TiMMCs) are made of a titanium alloy matrix reinforced with fibers, particles, or whiskers [3] which provide them exceptional properties such as a high specific modulus, high specific strength, high-temperature and wear resistance, and potential decreased weight. In the last 30 years, TiMMCs have emerged as an alternative to Ni-based alloys in aerospace applications [3,4,5]. They are suitable for high-performance applications such as rotating components of jet engine compressors [6]. However, the complexities of fabrication and high material and processing costs limit their wide industrial applications. Recent research efforts by NASA, made it possible to implement their use in large gas turbine engines [7,8] resulting in successful applications of TiMMCs. However, research efforts are still required on the precision machining of TiMMCs. TiMMCs reinforced with fibers are difficult and expensive to fabricate, industries prefer TiMMCs reinforced with particles or whiskers which have equivalent performances but without the disadvantages of anisotropic strength. The difference in mechanical properties between the matrix (ductile) and the particle reinforcements (brittle, hard, and abrasive), and the high strength and low heat transfer of TiMMCs present challenges during machining [9]. Therefore, TiMMCs parts are generally made near net shape to avoid heavy machining operations. The issues in finishing TiMMC parts include high tool wear [10] and poor surface finish with voids, micro-cracks, and broken particles. The surface defects constitute crack initiation sites rendering the workpieces vulnerable to fatigue resistance [11,12], which is essential for use in high-performance applications of TiMMCs.

The literature contains a substantial amount of information regarding the machining of metal matrix composites (MMCs). Almost all typical machining methods have been studied in the literature, whether it is a conventional or unconventional machining methods. The main tool wear mechanisms were identified as abrasive wear [13], flank wear, and built-up edge [14]. The predominant tool wear mechanism for machining MMCs is two-body abrasion and three-body abrasion [15]. The effectiveness of different tool materials for a multitude of MMCs with reinforcing particles was also studied. Polycrystalline diamond (PCD) tools are more suitable than polycrystalline cubic boron nitride (PCBN) or carbide-based tool materials [16,17,18]. In general, studies have shown that the tool life of cutting tools made with a material less hard than SiC particles is very limited [19]. Machining of MMCs has shown that cutting speed is the parameter with the greatest impact. Both tool wear and roughness surface are greatly influenced by cutting speed, in various studies [14,20,21,22,23,24,25]. Laser machining has no advantage for the machining of MMCs. The excessive heat produced during machining negatively affects the surface finish [26]. Even though TiMMCs are an integral part of MMCs, the substantial information regarding the machining of MMCs is mainly focused on aluminum alloy matrix MMCs with SiC particle reinforcements. Lately, there has been many activities on machining TiMMCs. The present paper provides an overview of the latest advances in machining of TiMMCs. It covers the operations of turning, milling, and grinding with focus on the effect of different cutting parameters, material removal mechanisms, tool wear, and the surface finish. Comparisons of the behavior during machining between MMCs and TiMMCs are also presented and discussed throughout the paper.

## 2. Turning

Certain facts demonstrated for MMCs are taken for granted as being true for TiMMCs. These facts are mainly the dependence of the turning efficiency on the workpiece material [27,28], the dependence of the surface quality on the tool material, grain size, and other aspects such as the effect of the depth of cut, feed rate [29], and cutting speed [15] on tool wear and surface quality. Most studies on the turning of TiMMCs address tool wear or the quality of the machined surfaces. The difficulties related to the machining of TiMMCs guided research efforts. Studies, which are sometimes less conventional, were conducted to find cutting conditions to minimize tool wear while increasing the quality of the surface finish during turning. This section discusses these topics and some characteristics for turning the TiMMCs.

**Figure 1:**
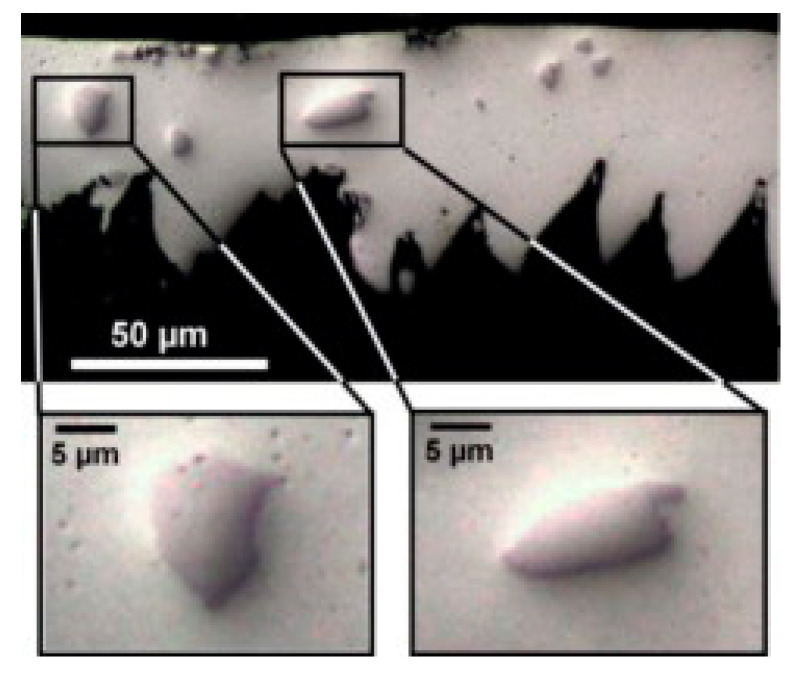
Unbroken particles at surface temperature 1000 °C and v_c_ = 100 m/min with laser-assisted machining [9].

The effect of workpiece temperature on the machining performance was studied. An improvement in surface roughness was obtained when an Al-based MMC was pre-heated to 60 °C [26]. For TiMMCs, a study was carried out on laser-assisted turning [9]. The laser increases the local temperature of the TiMMCs just before cutting. During the study, the temperature was maintained below the phase change temperature of the Ti_6_Al_4_V matrix (882 °C), between 300–500 °C. Although the surface roughness increases by 15%, the total volume of material removal and the tool life increased. The increase in tool life is due to the fewer number of broken particles (Figure 1) and consequently, less abrasion wear. A high temperature (500 °C) coupled with a high cutting speed (v_c_ = 170 m/min) can induce tool diffusion wear to the point that it becomes the source of the cutting tool wear. The increase in tool life during laser-assisted turning is accompanied by reduced cutting forces. The thrust force was reduced by around 25%. The variation in cutting forces are recognized as a significant indicator of tool wear in the machining process [14]. However, cutting temperature also has effect on tool wear.

Tool life is affected by the nature of the tool. It is shown in [20,21,22,23] that the tool life is increased for coated carbide tools used in MMC finishing compared to uncoated carbide tools. PCD tools are highly recommended in the literature for turning MMCs because they are harder than the particle reinforcements [14] and do not chemically react with the components of the part [16]. The effectiveness of coated tools on extending tool life applied to turning TiMMCs too. A comparative study conducted by Alireza [30] showed the effectiveness of different cutting tools for turning TiMMCs. The study compared the tools of tungsten carbide inserts coated with TiSiN-TiAlN, cubic boron nitride (CBN) inserts, and PCD inserts. The results showed that the carbide inserts provide a much better surface finish than the other two types of inserts. The use of CBN inserts for the same turning conditions leads to the surface being 150% rougher than carbide inserts: 2.29 µM versus 1.42 µM. The use of PCD inserts is not at all suitable for turning TiMMCs. An extremely high wear rate is observed leading to tool chipping and failure. An increase in cutting speed and depth of cut beyond 500 m/min and 0.5 mm/rev, leads to the catastrophic effect: The breakage of the insert. For other turning conditions (cutting speed between 50–120 m/min and a feed rate of 0.1 or 0.2 mm/rev) of the same TiMMCs, Bejjani [31] observed that using PCD tools led to less rough surfaces than using carbide tools, and that the surface roughness was more sensitive to the variation of cutting speed than feed rate. For high cutting speed conditions, the mechanisms change and provide a better surface finish. Moreover, beyond 170 m/min, turning can only be done with a PCD tool because the carbide tools wear rapidly [32]. Niknam et al. [33] compared carbide and CBN inserts. Their results showed that both types of inserts are subject to flank wear and the main wear mechanisms are abrasion and adhesion. The observation of a rapid increase of the cutting force from 120–500 N during turning at high speed (80 m/min) with carbide inserts revealed rapid tool wear and poor surface quality. CBN cutting tools seem to be the best choice for turning TiMMCs due to their high abrasion and resistance to thermal load.

In the literature [15,34], two- and three-body abrasion are found to be the dominant tool wear mechanisms for the MMCs. The presence of hard particles in TiMMCs leads to both two- and three-body abrasion wear. TiMMCs also exhibit strong chemical reactivity due to the chemical nature of the matrix (titanium alloy) which results in a strong affinity to almost all tool materials [35]. Adhesion is the major damage mechanism of the tool observed during the turning of TiMMCs by Alireza [30] for carbide and CBN inserts. Abrasion and diffusion are secondary wear mechanisms in this study. The tooltip is protected against abrasion due to the formation of a smooth layer of adhered material. For turning at high cutting speed, MMCs are very abrasive and tend to wear the tool rapidly [31,36]. For TiMMCs, a high cutting speed (170 m/min) tends to reduce tool wear. The increase in cutting speed above 180 m/min seems to lead to a change in the cutting mechanism causing a decrease in cutting force, and therefore a decrease in tool wear. At such a cutting speed, abrasion is the main tool wear mode. Laser-assisted turning is a good alternative to increase tool life. The tool life is linked to particle-tool interactions. When the matrix is heated by the laser (300−500 °C), the particles will move into the softer matrix instead of contributing to the abrasion wear of the tool. Even though abrasion wear mode remains the dominant wear mode, high cutting speed, and high temperature during laser-assisted turning induced diffusion wear mode at the tool chip interface. X.T. Duong et al. [37] reported that the initial tool wear mechanism would significantly affect the entire tool life when machining TiMMCs. Their subsequent study made it possible to highlight the formation of a “wear shield” on the surface of the cutting tool from the first moment of cutting. The formation of this wear shield is due to the adhesion of the tool on the workpiece (taking place at low cutting speeds), coupled with the diffusion wear (chemical reactivity of the matrix), and would make it possible to limit the tool wear during steady wear. Diffusion allows the creation of a new hard thin layer on the surface of the tool which will constitute the “wear shield”. The main wear modes observed during the initial tool wear are therefore adhesion and diffusion. The wear shield protects the tool against the abrasive effect of titanium carbide particles.

**Figure 2:**
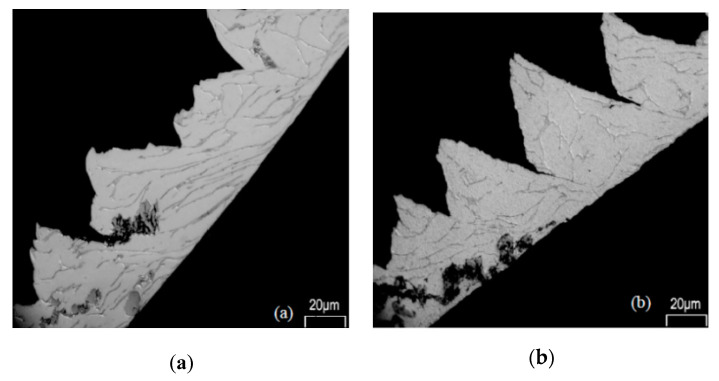
Chip microstructure with the polycrystalline diamond (PCD) tool at (**a**) 100 m/min; and (**b**) 230 m/min [32].

**Figure 3:**
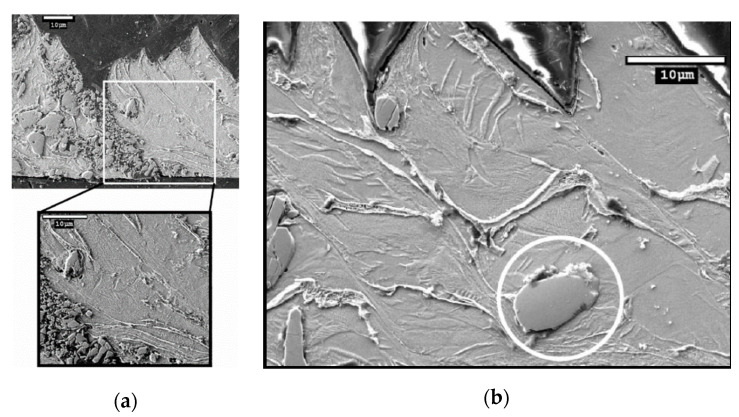
(**a**) Particle broken into many other smaller particles during the chip formation at v_c_ = 60 m/min; (**b**) Unbroken particle during the chip formation at v_c_ = 180 m/min [32].

**Figure 4:**
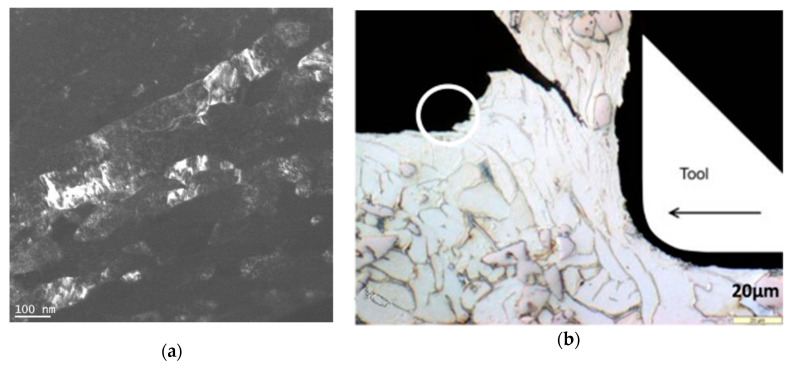
(**a**) Darkfield imaging of sub-grains inside the adiabatic shear band (ASB); (**b**) Sign of crack initiation at the free surface ahead of the tool (v_c_ = 60 m/min) [38].

The study of chip formation and particle-tool interactions clarifies the mechanisms that operate during machining. Bejjani et al. [31] investigated mechanisms due to the tool-chip interaction, and chip formation at different cutting speeds (from 60–230 m/min). The low thermal conductivity of titanium alloy matrices leads to adiabatic shear and results in segmented chips. Chip formation at different cutting speeds does not occur in the same way. At low speed (100 m/min) Bejjani et al. showed that the chip microstructure had undergone high plastic deformation, with a plastic strain over most of the area of the chip (Figure 2). The presence of these high stresses is sufficient to break the particles, resulting in the formation of a multitude of small particles which contribute to the increase of the tool wear rate (Figure 3). While at high speed (230 m/min) the microstructure shows smaller deformations, demonstrated by a larger shear angle. At these cutting speeds, particles are displaced or cut and result in less abrasion wear of the tool. The study also shows that at different cutting speeds there was no evidence of particle de-bonding. In a subsequent study, Bejjani et al. [38] investigated the nature of chip segmentation and tool-particle interactions to confirm the mechanisms reported above. The observation of the chip formation revealed a decrease in the segmentation of the chip with an increase in the cutting speed: Respectively, 150 µM and 90 µM at 50 and 150 m/min. The segmentation mechanism was observed to start from a crack on the free surface material ahead of the tool (Figure 4b). The transmission electron microscopy (TEM) and scanning transmission electron microscopy (STEM) studies revealed that there is an evolution of the microstructure in the adiabatic shear band (ASB) producing sub-grains (Figure 4a). These sub-grains exhibit misorientations characteristic of rotational dynamic recrystallization (RDX). Calculations show that the temperature in the ASB is close to the recrystallization temperature (500–690 °C). A microstructural evolution model of the chip is proposed for the first time. Otherwise, the particles present in the matrix do not interfere with the formation of the ASB.

Dust generation during machining is a health and environmental concern that received very little attention in the current global context. Machining TiMMCs produces severe tool wear and probably a strong particulate emission. A recent study [39] reported the dust generation at micro- and nano-scales under different lubricated modes during machining of TiMMCs. The cutting speed shows a significant impact on the production of fine particles (2.5 µM aerodynamic particles diameter or less) while it does not affect the specific surface concentration and mass concentration of ultrafine particles (size range of 0.1 µM). As expected, tool lubrication reduces particle emission during turning, and high levels of flow rate (300 mL/min) combined with coated inserts lead to less ultrafine particles than with uncoated inserts. Under dry conditions, particle emission is more important, but the analysis of the cutting forces shows that at a high flow rate (300 mL/min) there is a thin film of lubricant formed. The presence of this thin film of lubricant may affect the emission of particles under high lubrication modes and high speeds (50 m/min). Finally, even if the dry condition provides a smaller roughness (around 1 µM, versus 3 µM for a flow rate of 300 mL/min) [40], the emission of ultrafine particles and fine particles with a coated tool remains significant compared to lubricated conditions.

## 3. Milling

Also, the numbers of publications on conventional milling of TiMMCs is fewer than turning, comparison can still be done between the different mechanisms occurring during the milling of TiMMCs and those of MMCs. As with turning, tool wear and surface roughness are the main concerns in milling TiMMCs. The studies available in the literature provide a comprehensive comparison between different tool materials during high-speed milling [30,41]. The tools compared are two carbide tools (X400 and X500) and a PCD tool. Studies on the milling of MMCs have shown that carbide tools are not very effective and have lower tool life than PCD tools [17,18]. For the milling of TiMMCs [30,41], the carbide tools demonstrated acceptable tool wear despite the occurrence of vibration. Similar to PCD tools, carbide tools have shown the possibility of an optimum tool wear and productivity under certain conditions. These optimum conditions are v_c_ = 45 m/min, f = 0.2 mm/rev, a_p_ = 0.16 mm for the X500 carbide tool, and v_c_ = 70 m/min, f = 0.35 mm/rev, a_p_ = 1.5 mm for the X400 carbide tool. The observations made during the study showed that cutting speed is the main factor influencing the tool wear rate for the PCD tool. Optimum tool wear is obtained at low cutting speeds (150 m/min). It is important to note that in the cutting speed range of 350−600 m/min, PCD tools failed. The tool wear of the carbide tools is equally highly dependent on the cutting speed, as well as the depth of cut. These two parameters govern the tool wear rate during milling with carbide tools. These observations do not match those made for the milling of MMCs. Indeed, studies have shown that the feed rate is the cutting parameter that significantly affects the tool wear [42,43]. Although the cutting conditions for both types of tools used for milling TiMMCs are similar, the wear mechanisms are not identical. Tool wear mechanisms vary depending on the type of tool. Observations showed the presence of oxidation on the edge of the carbide inserts, but adhesion was the main wear mechanism. While for PCD tools, the adhesion was very slight and the main wear mechanisms were identified as edge chipping, abrasion, and peeling.

As surface roughness is a primary concern in machining TiMMCs, cutting parameters with considerable influence on roughness were investigated. For carbide tools, statistical analyses have shown that the feed rate is the parameter having the most influence on roughness [41]. A feed rate of 0.2 mm/rev permits to obtain a roughness of 1.74 µM for a X500 carbide tool. Similarly, for milling of MMCs, the feed rate is likewise one of the cutting parameters having the most impact on roughness [24,25]. PCD tools for milling TiMMCs do not show any dependence on one specific cutting parameter, but rather on a set of different cutting parameters. Optimization of these cutting parameters is necessary to obtain a quality surface roughness. As an example, a roughness of 0.18 µM can be achieved after optimization of the parameters. An investigation on the milling of a TiMMCs reinforced with carbon nanotubes was conducted. It was found that the roughness is lower at high cutting speed (v_c_ = 188.4 m/min) and low feed rate (200 mm/min). Unfortunately, the cutting tool material was not provided, which makes it impossible to compare with other investigations [44].

**Figure 5:**
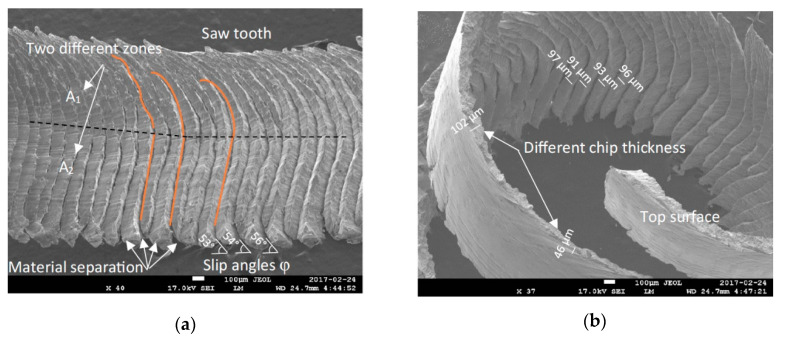
(**a**) two distinct zones with two different compression ratios; (**b**) different shear slip distances [41].

Chip morphology study is important in understanding the mechanisms that operate during milling just as it is in turning. In general, the study conducted by Kamalizadeh et al. [41] shows that the chips obtained during milling of TiMMCs are similar to those obtained during turning: Serrated chips. Unlike chips formed during turning, milling chips have two different chip compression ratios, thus creating two main zones. The former is the A1 zone and the later the A2 zone as shown in Figure 5a. These zones are due to either the non-linear movement of chips caused by catastrophic shearing during the segment formation (A2) or the high temperature at the tool chip interface (A1). Statistical studies conducted on the shear slip distance and slip angle show that they increase slightly with the time (Figure 5b). The authors attribute this phenomenon to a serration level which becomes more and more intense with time. This is closely linked to the increase in cutting force, friction force, as well as the rapid increase in temperature in the shearing bands characteristic of machining TiMMCs.

## 4. Grinding

The exceptional properties of TiMMCs make them good candidates for manufacturing parts for the aerospace industry, especially for large gas turbine engines [7,8]. However, the reinforcing particles in the TiMMCs cause the defects on machined surfaces. These defects are the seat of the formation of fatigue cracks under repeated loading, leading to the fracture of the part. The fatigue resistance of aeronautical parts is essential to avoid catastrophic accidents. The surface finish is one of the main concerns for TiMMCs research. As the grinding is mainly used to increase the accuracy of the workpiece and surface quality [45,46], understanding the mechanisms operating during grinding is essential.

Several previous studies [47,48,49,50] showed that grinding forces affect roughness, work hardening, power consumption, grinding stresses, and surface defects, and give a qualitative indication of the tool wear and grindability (the ease of removal material). For MMCs, the literature has shown [51] that grinding wheel wear increases with increasing contents of the reinforcing particle because the particles are highly abrasive. In their study on TiMMC, Blau et al. [49] showed the influence of particle content on grinding parameters, including grinding forces. The results showed that when the content of TiC particles increases from 0% to 10%, the grinding force increases as does the resistance to material removal, and therefore the tool wear. The results follow the same trend as those obtained for MMCs [52,53].

Depth of cut is the grinding parameter having the most influence on cutting forces during grinding TiMMCs [54]. Comparisons of cutting force in high-speed grinding (120 m/s) and conventional-speed grinding (20 m/s) have shown that cutting forces decrease with increasing cutting speed [54,55]. Even though cutting speed is not the most influential cutting parameter, it has a significant influence on cutting forces. Different types of defects such as microcracks and pile-ups can influence roughness; in particular, through the grinding scratches and cavities related to the ductile deformation of the matrix and the fragile behavior of the reinforcing particles [55]. The grinding forces [56] and the size of the undeformed chip thickness [55] play an essential role in the propagation of microcracks, in particular lateral crack propagation. The presence of microcracks on the ground surface is due to severe residual stresses from the large mechanical-thermal loads occurring during grinding [57]. Zheng Li et al. [55] compared the ground surface morphology obtained in conventional-speed grinding (20 m/s) and high-speed grinding (120 m/s). They observed that grinding scratches and cavities are related to the ductile deformation of the matrix and the fragile behavior of the reinforcing particles. The formation of pile-ups is due to the slide flow of the material during grinding. High-speed grinding induces a decrease in the presence of pile-up thanks to the increase of active grains per unit of time. The size of the pile-up is highly dependent on the depth of cut or on the undeformed chip thickness, which is consistent with previous studies [58,59,60] and studies on MMCs [61].

The content of reinforcing particles in the composite also has an influence on the roughness of ground surfaces. The surface roughness of MMCs increases with the content of reinforcing particles [52]. However, the roughness for the TiMMCs decreases [49]. It can be assumed that the difference in roughness tendency is due to the nature of the particles and the matrix. TiC particles are harder than SiC particles, and Ti_6_ Al_4_V is more ductile compared to an Al alloy. During grinding, SiC particles can easily break up into a multitude of small particles that become encrusted in the ground surface of the Al alloy, increasing the roughness. Ti_6_Al_4_V is more difficult to machine due to its high ductility. Without particles, its ductility can induce the formation of pile-ups as well as numerous grooves. The presence of TiC particles limits the formations of pile-ups and grooves. The very hard nature of the TiC particles seems to promote the dislodgement of the particles instead of their breaking, which is favorable to the reduction in roughness. Table 1 presents some of the roughness measurement data from articles of interest to illustrate the point.

**Figure 6:**
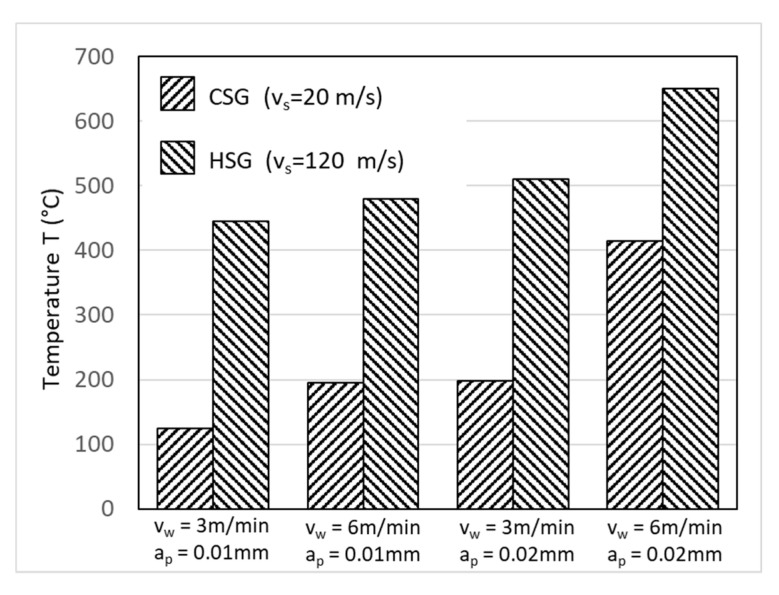
Effects of grinding parameter in high (HSG) and conventional (CSG) speed grinding on grinding temperature [55].

One might think, instinctively, that the grinding temperature depends on the grinding forces. However, Zheng Li et al. [54,55] showed that the same cutting parameters influence the grinding temperature and the grinding forces, but in a different way. When the grinding speed increases from 20–120 m/s, the grinding forces decrease by up to 50% but the grinding temperature increases. The authors attributed the decrease in grinding force to the lower friction coefficient in high-speed grinding, which is questionable. Conversely, the grinding temperature increase is caused by the increased action of the grinding grains in the contact zone per unit of time coupled with the drop-in efficiency of the coolant. The drop-in efficiency of the coolant is due to an air barrier that forms around the abrasive wheel, thus preventing the cooling fluid from entering the grinding zone leading to very poor efficiency of the coolant and at an increase in the grinding temperature. The effect of the different grinding parameters on the grinding temperature is shown in Figure 6. Different from grinding speed, the parameters of workpiece speed and the depth of cut have the same effect on both grinding forces and grinding temperature.

**Figure 7:**
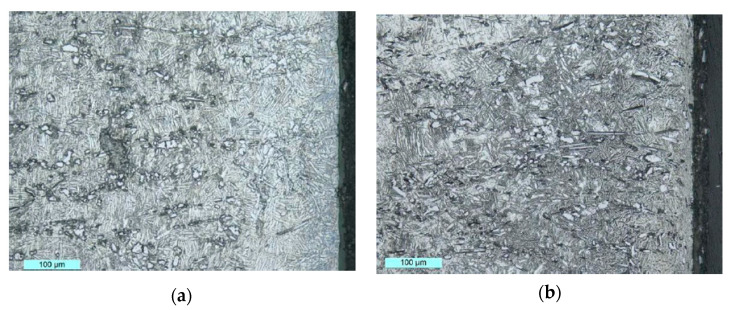
Changes in microstructure: (**a**) v_s_ = 25 m/s, v_w_ = 300 mm/s, a_p_ = 0.3 mm; (**b**) v_s_ = 20 m/s, vw = 300 mm/s, a_p_ = 1.0 mm (v_s_ = cutting speed, v_w_ = speed of the workpiece, and a_p_ = cutting depth) [62].

Temperature variations during grinding can have significant impacts on the microstructural or mechanical integrity of the material. Large temperature variations can lead to the presence of residual stresses on the subsurface as well as microstructural transformations [62,63,64]. The presence of micro-cracks on the ground surface is characteristic of severe residual stresses [57]. Zheng Li et al. [62] observed residual compressive stress, work hardening on the ground surface, and microstructural changes in the subsurface. The microstructural changes observed are the bending and elongation of the crystal lattice as shown in Figure 7. These changes are mainly related to the strong plastic deformations and rapid cooling effects that occur on the surface during grinding. The authors showed that the depth of cut is the parameter that induces the higher variation in the microstructure.

Microstructural changes were also observed in the study by Xinxin Xi et al. [65]. Microstructural changes were highly dependent on the same grinding parameter, or the depth of cut. When the depth of cut is 0.020 mm, the distinction of the grain boundary is difficult due to the microstructural changes (up to a thickness of about 20 µM), when the depth of cut is 0.002 mm the microstructure is slightly altered (up to 5 µM) deep below the surface. Microhardness tests were also carried out by the authors to validate their microstructural observations. The results showed that microhardness increases (from 250–425 HV) with the decreasing depth below the ground surface (from 225–10 µM, respectively). This increase in hardness reflects significant residual stresses and/or a microstructural change linked to grinding.

Similar to turning and milling, the understanding of chip morphology and the material removal mechanisms in grinding is important for the optimization of grinding parameters. Ding et al. [56] studied the material removal behavior. Their results show that the material removal behavior is divided into three stages. Stages one and three belong to ductile regime removal of the matrix, dominated by serrated chip formation. Meanwhile, stage two belongs to the brittle regime removal dominated by brittle fracture, crack initiation, and crack propagation. The authors extended their study to investigate the effect of grinding speed on material removal mechanisms. The results show that the chip morphology varies with the grinding speed. The chip morphology is continuous and thicker at the speed of 20 m/s. It becomes discontinuous and thinner at 120 m/s. At high-speed grinding (120 m/s), the material removal behavior is dominated by a high strain and strain-rate hardening which induces a weakened and brittle plastic flow. Similar results were obtained by Xinxin Xi et al. [65]. Specifically, the material removals behavior observed are the same, namely: The ductile removal of the matrix, ductile removal of the particle and brittle behavior, fracture, and crack propagation for the particles. Moreover, these different stages of material removal mechanism are the same as those observed for MMCs in the study conducted by Huang et al. [66].

**Figure 8:**
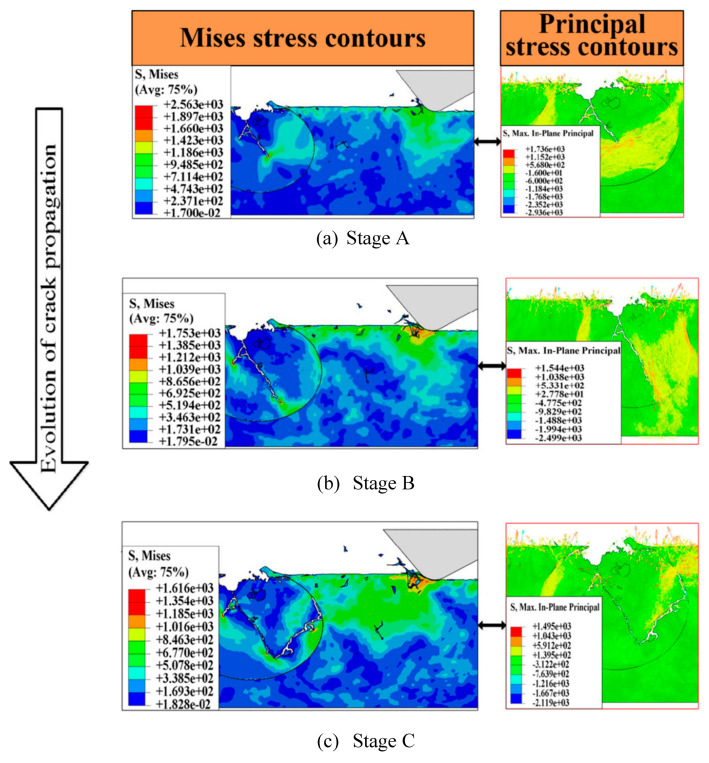
Evolution of the crack propagation for the second grinding grain. (**a**) initial stage, (**b**) developing and (**c**) complete [67].

**Figure 9:**
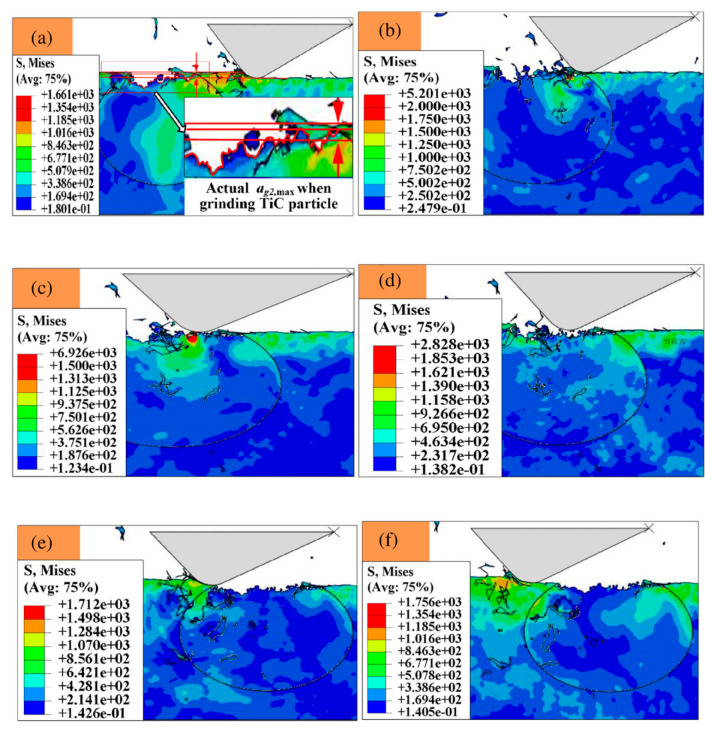
Material removal mechanism details from stage (**a**) to (**f**); (**a**) grinding the matrix, (**b**) the initial contact between the CBN grain and the TiC particle, (**c**) initial grinding of the particle, (**d**) particle removal process, (**e**) particle failure completed, (**f**) grinding the matrix [67]

Most studies on material removal mechanisms of grinding are based on experimentally validated finite element (FE) models. The work of Huan et al. [67] modeled for the first time the simultaneous action of two abrasive grains of a CBN wheel in high-speed grinding (120−140 m/s). All relevant details of the material removal behavior during grinding are developed. During the grinding by the first grain of CBN, there are two possible outcomes: Either the TiC reinforcement has a residual crack, or it does not. In the case of a residual crack during the passage of the second grain, the FE simulation showed that the maximum stress of Mises was located at the point of the crack in the reinforcement particle, regardless of the distance between the grain of the grinding wheel and the interface. The residual crack spreads during the passage of the second grain of the wheel until the tensile failure of the TiC particle. Then the reinforcement particle breaks off the matrix. During these conditions, stress concentrations are present but do not continuously exchange between the matrix and the reinforcement. These mechanisms are illustrated in Figure 8. In the case of no residual crack, there is no stress concentration which leads to the tensile failure of the particle. The position of the maximum Mises strain is located at the point of contact between the grain of the wheel and the workpiece. The grinding action of the second grain therefore causes the plastic removal of the matrix as well as the brittle removal of the TiC particle. Figure 9 shows a schematic of the mechanism. The results also show that for the passage of the first grain of the wheel that the crushing depth is affected by the undeformed chip thickness. The more the undeformed chip thickness increases, the more the crushing depth of the particles increases in high speed grinding.

**Figure 10:**
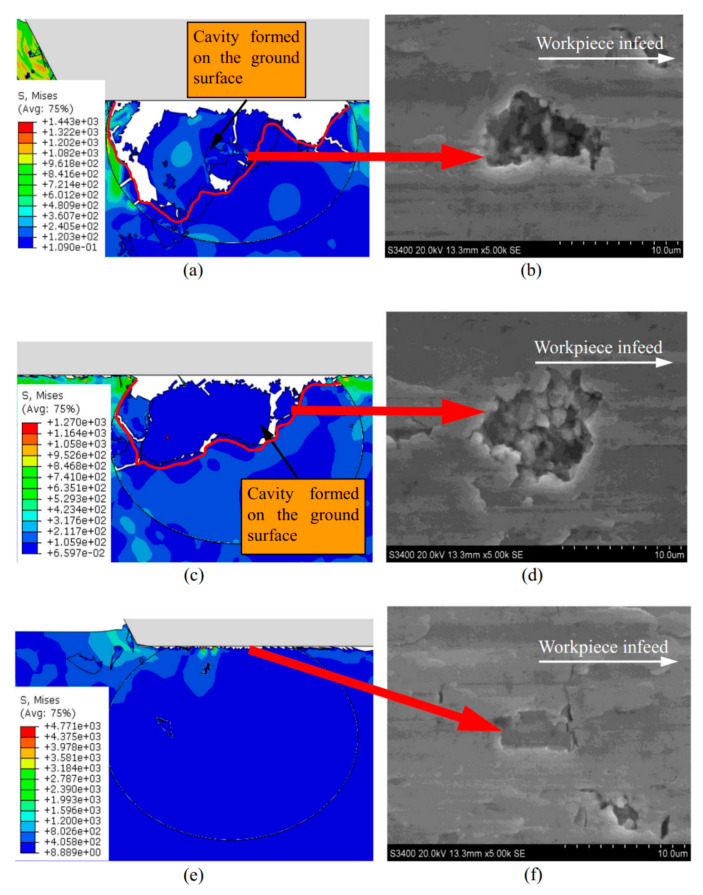
Effect of undeformed chip thickness on ground surface. Experiment versus simulation. (**a**) and (**b**) a_p_ = 1.2 µM, (**c**) and (**d**) a_p_ = 0.7 µM, and (**e**) and (**f**) a_p_ = 0.2 µM [65].

Other studies have shown that the undeformed chip thickness influences many parameters, notably the grinding force, grinding temperature, surface roughness, and material removal mechanism [55,56,57]. The trend of the results obtained showed that the grinding forces decrease and that the grinding temperature increases with the decrease in the undeformed chip thickness. Observations of the surfaces after grinding revealed that when a small undeformed chip thickness (0.3 µM) is employed, the particles are subject to a tiny fracture which generates a small and shallow cavity on the ground surface. When the undeformed chip thickness is large (1.2 µM), the cavities of the ground surface are deep and wide. These differences in material removal mechanism result in the differences in the ground surface roughness. A wide undeformed chip thickness will increase surface roughness [55]. Besides, they observed that the lateral crack formation is dependent of the undeformed chip thickness. The larger the undeformed chip thickness, the deeper the depth of the lateral crack becomes. A deeper depth of the lateral crack leads to an increase of the interactions between cracks which can cause them to emerge on the ground surface and alter the roughness [55]. The influence of the undeformed chip thickness on the depth of the voids left by the fragile rupture of the particles was also observed by Xinxin et al. [65]. Their study in high-speed grinding showed that the bigger the undeformed chip thickness, the larger and deeper the cavity produced on the ground surface. The results of the finite element modeling were verified experimentally for similar grinding conditions and presented in Figure 10.

Ding et al. [50] employed the undeformed chip thickness to study the specific grinding energy which is a fundamental parameter for the characterization of the grinding process. The results showed that the specific grinding energy is slightly smaller for the TiMMC compared to Ti_6_Al_4_V (around 20 J/mm^3^ lower). They highlighted the fact that the specific grinding energy is dependent on the mechanical properties of the material under grinding conditions [68]. It is well known in the literature that the complete ductile-mode removal of the material during the grinding requires a higher specific grinding energy compared to brittle-mode removal. As presented by Malkin et al. [69], when brittle fracture takes place during grinding, the specific grinding energy is lower than that for ductile behavior. The presence of brittle breaking of the reinforcements in the TiMMCs explains the observations made by the authors. The results also showed that the more the undeformed chip thickness increases, the more the specific grinding energy decreases. This is explained by the dependence of specific grinding energy on the following parameters: Energies consumed in chip formation, elastic deformation of the workpiece, sliding, and plowing [50,69].

The grinding wheel performance in terms of wheel life, grinding performance (forces, temperature, energy), and ground surface finish is one of the main concerns in machining TiMMCs. Zheng Li et al. [62] compared three types of conventional wheels: White alumina (WA), pink fused alumina (PA), and micro-crystal corundum (SG) grinding wheels. The SG wheel showed better performance, with the lowest grinding forces and force ratio, grinding temperature, specific energy, and surface roughness. The ground surface obtained with the SG wheel contains only a few voids and slight grooves, while the other two wheels which presented voids, debris, adherence, grooves, and delamination zone. The grinding temperature is closely related to the thermal conductivity of the grinding wheel. Indeed, Xin-Xin Xi et al. [70] compared the heat transfer characteristic of electroplated CBN wheel and vitrified CBN wheel for high-speed grinding. It was shown that the grinding temperature was lower with an electroplated CBN wheel due to its high thermal conductivity. Other authors [57] compared the performance of wheels on TiMMCs with randomly distributed grains and linearly organized grains (spaced 1.2 mm apart). The former is a single layer electroplated wheel and the latter is a brazed CBN wheel. The brazed wheel showed a removal rate twice as high as that of the electroplated wheel, it had the lowest grinding forces, grinding temperature, and less ground surface defects as well. The brazed wheel showed a better potential for high-speed grinding, without burning at the ground surface.

## 5. Conclusions

This paper presents a critical review on the machining of TiMMCs with focus on tool wear mechanisms, material removal and chip formation mechanisms, and machined surface quality for turning, milling, and grinding operations. The following conclusions can be drawn:

- Machining of TiMMCs presents challenges due to the presence of hard abrasive particles in a softer and sticky matrix and the low thermal conductivity of the matrix material. TiMMCs are significantly different from other types of MMCs. The findings from the machining of other MMCs cannot be taken for granted for machining of TiMMCs.
- The information in the literature about the turning of TiMMCs is quite comprehensive and the mechanisms that operate during the process are well understood. However, research efforts still need to be made regarding the emission of particles. In the milling and grinding of TiMMCs, the literature is very limited. There is a bright future for further investigations.
- For turning operations, tool wear tends to decrease in high-speed turning. No evidence of particle de-bonding was observed in turning of TiMMCs regardless of the cutting speed. The segmentation of the chip starts at the free surface ahead of the tool. The tool wear mechanisms are dependent on the cutting speed. Adhesion wear is the main wear mode and abrasion, and diffusion are secondary mode in conventional speed turning. Abrasion wear is the main wear mode in high speed turning of TiMMCs. The formation of a “wear shield” on the surface of the cutting tool from the first moment of cutting and under specific conditions results in enhanced tool life.
- For milling operations, surface roughness dependency on the cutting parameters varies with the cutting tool. The main wear mode is dependent on the cutting tool.
- For grinding, wheel wear increases with increasing contents of the reinforcing particle for both TiMMCs and MMCs. Surface roughness, however, decreases with the increase of in the percentage of hard particles. The undeformed chip thickness is the most important parameter which affects the material removal mechanisms, ground surface microstructure, lateral cracks, surface cavities, and surface roughness.

In the future, to find the effect on grinding or milling condition on tool wear would be of major interest. The study of the efficiency of other cutting tools such as diamond tools could provide interesting insights in terms of TiMMCs machinability. Dust emission is also a field of study which should not be left aside in order to comply with occupational safety standards and to limit the pollution resulting from the processing of TIMMCs.

## Figures and Tables

**Table 1:** Effect of particles content on roughness.

	Blau et al. [49]		Thiagarajan et al. [52]	
Particle %	Ti_6_Al_4_V + 0% TiC	Ti_6_Al_4_V + 10% TiC	Al alloy + 2% SiC	Al alloy + 12% SiC
Ra (µM)	1.68	0.86	0.20	0.60
